# Prognostic value of non‐resistant and resistant masked uncontrolled hypertension detected by ambulatory blood pressure monitoring

**DOI:** 10.1111/jch.14460

**Published:** 2022-03-17

**Authors:** Francesca Coccina, Anna M. Pierdomenico, Chiara Cuccurullo, Jacopo Pizzicannella, Maria T. Guagnano, Giulia Renda, Oriana Trubiani, Francesco Cipollone, Sante D. Pierdomenico

**Affiliations:** ^1^ Department of Innovative Technologies in Medicine & Dentistry University “Gabriele d'Annunzio”, Chieti‐Pescara Chieti Italy; ^2^ Department of Medicine and Aging Sciences University “Gabriele d'Annunzio”, Chieti‐Pescara Chieti Italy; ^3^ Department of Neurosciences, Imaging and Clinical Sciences University “Gabriele d'Annunzio”, Chieti‐Pescara Chieti Italy

**Keywords:** blood pressure, classification, hypertension, masked hypertension, risk

## Abstract

Masked uncontrolled hypertension (MUCH) is at higher cardiovascular risk than controlled hypertension (CH). In previous studies, patients with MUCH were considered as a unique group though those receiving ≤2 drugs could be defined as having nonresistant MUCH (NRMUCH) and those receiving ≥3 drugs as having resistant MUCH (RMUCH). The aim of this study was to assess the prognostic value of NRMUCH and RMUCH detected by ambulatory blood pressure (BP) monitoring. Cardiovascular risk was evaluated in 738 treated hypertensive patients with normal clinic BP. Patients were classified as having CH or MUCH if daytime BP < or ≥ 135/85 mmHg, respectively, regardless of nighttime BP, or CH or MUCH if 24‐h BP < or ≥ 130/80 mmHg, respectively, regardless of daytime or nighttime BP. By daytime or 24‐h BP, the authors detected 523 (71%), 178 (24%), and 37 (5%) or 463 (63%), 231 (31%), and 44 (6%) patients with CH, NRMUCH, and RMUCH, respectively. During the follow‐up (median 10 years), 148 events occurred. After adjustment for covariates, compared to CH, the hazard ratio (HR), 95% confidence interval (CI), for cardiovascular events was 1.81, 1.27–2.57, and 2.99, 1.73–5.16, in NRMUCH and RMUCH defined by daytime BP, respectively, and 1.58, 1.12–2.23, and 2.21, 1.27–3.82, in NRMUCH and RMUCH defined by 24‐h BP, respectively. If RMUCH was compared with NRMUCH, the risk tended to be higher in RMUCH but did not attain statistical significance (*P* = .08 and *P* = .23 by daytime and 24‐h BP thresholds, respectively). In conclusion, both NRMUCH and RMUCH are at increased cardiovascular risk than CH.

## 1 INTRODUCTION

Masked uncontrolled hypertension (MUCH), that is, normal clinic but high out‐of‐office blood pressure (BP) in treated patients has been widely studied in the latest years.^1^, ^2^, ^3^, ^4^, ^5^, ^6^, ^7^, ^8^, ^9^, ^10^, ^11^, ^12^, ^13^, ^14^, ^15^, ^16^ Single studies and meta‐analyses have shown that MUCH is at higher cardiovascular risk than controlled hypertension (CH), that is, normal clinic and out‐of‐office BP.^1^, ^2^, ^3^, ^4^, ^5^, ^6^, ^7^, ^8^, ^9^, ^10^, ^11^, ^13^, ^14^, ^15^, ^16^

MUCH can be detected by using either home BP recording^1^, ^2^, ^3^, ^13^, ^14^, ^15^, ^16^ or ambulatory BP monitoring,^4^, ^5^, ^6^, ^7^, ^8^, ^9^, ^10^, ^11^, ^12^, ^13^, ^14^, ^15^, ^16^ according to which it is defined as home BP ≥ 135/85 mmHg or as daytime ≥135/85 mmHg and/or nighttime ≥120/70 mmHg and/or 24‐h BP ≥130/80 mmHg, respectively.

In previous studies, included patients with MUCH were treated with a different number of antihypertensive drugs.^1^, ^2^, ^3^, ^4^, ^5^, ^6^, ^7^, ^8^, ^9^, ^10^, ^11^, ^12^, ^13^, ^14^, ^15^, ^16^ In this scenario, those receiving ≤2 drugs could be defined as having nonresistant MUCH (NRMUCH) and those receiving ≥3 drugs as having resistant MUCH (RMUCH), in accordance with guidelines definition of resistant hypertension.^17^, ^18^ So far, previous investigations have evaluated the prognostic impact of MUCH considered as a unique group.^1^, ^2^, ^3^, ^4^, ^5^, ^6^, ^7^, ^8^, ^9^, ^10^, ^11^, ^13^, ^14^, ^15^, ^16^ Recently, an interesting study evaluated the prognostic value of RMUCH.^19^ The study was performed by using home BP recording and could not include NRMUCH.^19^ In such a context, other studies could be helpful to better understand the prognostic impact of MUCH subtypes.

The aim of this study was to evaluate the prognostic value of NRMUCH and RMUCH detected by ambulatory BP monitoring.

## 2 METHODS

### 2.1 Patients

We studied 738 treated hypertensive patients with normal clinic BP selected from 2264 sequential treated individuals aged 30–90 years who were prospectively recruited from December 1992 to December 2012. All these patients had been referred to our hospital outpatient clinic for evaluation of BP control. One hundred and three patients were lost during follow‐up. Patients with secondary hypertension were excluded. All the Patients underwent clinical evaluation, electrocardiogram, routine laboratory tests, echocardiographic examination, and noninvasive ambulatory BP monitoring. Study population came from the same geographical area (Chieti and Pescara, Abruzzo, Italy). The study was in accordance with the Second Declaration of Helsinki and was approved by the institutional review committee. Patients gave informed consent.

### 2.2 Clinic BP measurement

Clinic BP was recorded by a physician using a mercury sphygmomanometer and appropriate‐sized cuffs. Measurements were performed in triplicate, 2 min apart, at least after 5 min of rest and the mean value was used as the BP for the visit. Clinic systolic and diastolic BP were defined as normal when <140/90 mmHg.

### 2.3 Ambulatory BP monitoring

Ambulatory BP monitoring was performed with a noninvasive recorder (SpaceLabs 90207, Redmond, WA) on a typical day, within 1 week from clinic visit. Technical aspects have been previously reported.^20^ We evaluated the following ambulatory BP parameters: daytime (awake period as reported in the diary), nighttime (asleep period as reported in the diary), and 24‐h systolic and diastolic BP. MUCH was defined as clinic BP < 140/90 mmHg and two ambulatory BP definitions: (1) daytime BP ≥135 and/or ≥85 mmHg regardless of nighttime BP, (2) 24‐h BP ≥ 130 and/or ≥80 mmHg regardless of daytime or nighttime BP. All the patients had recordings of good quality (at least 70% of valid readings during the 24‐h period, at least 20 valid readings while awake with at least 2 valid readings per hour and at least 7 valid readings while asleep with at least 1 valid reading per hour), in line with the European Society of Hypertension requirements.^21^

### 2.4 Echocardiography

Patients underwent a comprehensive echocardiographic investigation, which included two‐dimensional, M‐mode, and Doppler examinations. Left atrial (LA) and left ventricular (LV) measurements and calculation of LV mass were made according to standardized methods.^22^ LA diameter (cm) was indexed by body surface area (m^2^) and LA enlargement was defined as LA diameter/body surface area ≥2.4 cm/m^2^.^22^ LV mass was indexed by height^2.7^ and LV hypertrophy was defined as LV mass/height^2.7^ >50 g/m^2.7^ in men and >47 g/m^2.7^ in women.^23^ LV ejection fraction (EF) was calculated using the Teichholz formula or the Simpson rule^22^ and defined as low when it was <50%.

### 2.5 Follow‐up

Patients were followed‐up in our outpatient clinic or by their family doctors. The occurrence of events was recorded during follow‐up visits or by telephone interview of the family doctor or the patient or a family member, followed by a visit if the patient was alive. Medical records were obtained to confirm the events. We evaluated a combined endpoint including coronary events (sudden death, fatal and nonfatal myocardial infarction, and coronary revascularization), fatal and nonfatal stroke, heart failure requiring hospitalization and peripheral revascularization. Outcomes were defined according to standard criteria as previously reported.^24^, ^25^, ^26^, ^27^, ^28^

### 2.6 Statistical analysis

Data are means ± standard deviation or numbers and percentages. Comparison between CH, NRMUCH, and RMUCH according to different definitions was performed by using one‐way ANOVA, followed by a multiple comparison test, for continuous variables and the chi‐square or Fisher's exact test, with Bonferroni's correction, for categorical variables. Event rates were expressed as the number of events per 100 patient‐years. Survival curves were estimated using the Kaplan–Meier product‐limit method and compared by the log‐rank test. Univariate and multivariate Cox regression analyses were used to estimate cardiovascular risk in patients with NRMUCH and RMUCH in comparison with CH, and between patients with RMUCH and NRMUCH. The forced entry model was used in multivariate analysis. Statistical significance was defined as *P* < .05. Analyses were made with the SPSS 21 software package (SPSS Inc. Chicago, IL).

## 3 RESULTS

Characteristics of patients with CH, NRMUCH, and RMUCH by daytime and 24‐h BP thresholds are presented in Table 1. Prevalence of men was higher in patients with NRMUCH and RMUCH than in those with CH. Body mass index and prevalence of LV hypertrophy were higher in patients with RMUCH than in the other groups. Prevalence of smokers was higher in patients with NRMUCH than in those with CH.

**TABLE 1:** Characteristics of patients

	Daytime BP threshold (regardless of nighttime BP)			24‐h BP threshold (regardless of daytime or nighttime BP)		
Parameter	CH	NRMUCH	RMUCH	CH	NRMUCH	RMUCH
n.	523	178	37	463	231	44
Age, years	61 ± 10	59 ± 11	62 ± 12	61 ± 10	59 ± 11	61 ± 12
Men, *n* (%)	202 (39)	123 (58)*	23 (62)*	166 (36)	134 (58)*	28 (64)*
Body mass index, kg/m^2^	28 ± 5	28 ± 4.0	30 ± 4*^†^	28 ± 5	28 ± 4	30 ± 4*^†^
Smokers, *n* (%)	84 (16)	48 (27)*	6 (16)	76 (16)	55 (24)	7 (16)
FHCVD, *n* (%)	64 (12)	17 (10)	2 (5)	59 (13)	22 (9)	2 (4)
Previous events, *n* (%)	29 (6)	7 (4)	1 (3)	24 (5)	12 (5)	1 (2)
Diabetes, *n* (%)	27 (5)	13 (7)	1 (3)	24 (5)	15 (6)	2 (4)
eGFR < 60 mL/min, *n* (%)	123 (23)	32 (18)	11 (30)	113 (24)	41 (18)	12 (27)
LDL cholesterol, mg/dl	129 ± 30	128 ± 28	119 ± 31	130 ± 29	127 ± 31	119 ± 27
LV hypertrophy, *n* (%)	79 (15)	39 (22)	18 (49)*^†^	69 (15)	47 (20)	20 (45)*^†^
LA enlargement, *n* (%)	74 (14)	28 (16)	4 (11)	62 (13)	39 (17)	5 (11)
ALVSD, *n* (%)	12 (2)	5 (3)	1 (3)	11 (2)	6 (3)	1 (2)

ALVSD, asymptomatic left ventricular systolic dysfunction (ejection fraction < 50%); BP, blood pressure; CH, controlled hypertension (below threshold value for each classification); eGFR, estimated glomerular filtration rate; FHCVD, family history of cardiovascular disease; LDL, low‐density lipoprotein; LA, left atrial; LV, left ventricular; NRMUCH, nonresistant masked uncontrolled hypertension (above threshold value for each classification); RMUCH, resistant masked uncontrolled hypertension (above threshold value for each classification).

* *P* < .05 versus CH and ^†^ *P* < .05 versus NRMUCH for each classification.

BP values are reported in Table 2. Though in the normal range, clinic BP was higher in patients with NRMUCH and RMUCH than in those with CH. Daytime, nighttime, and 24‐h BP were higher in patients with NRMUCH and RMUCH by definition. Clinic, daytime, and 24‐h systolic/diastolic BP, and nighttime diastolic BP were not different between patients with NRMUCH and RMUCH for each definition. Nighttime systolic BP tended to be higher, but did not attain statistical significance, in patients with RMUCH (*P* = .07 and *P* = .12 for daytime and 24‐h definitions, respectively).

**TABLE 2:** Blood pressure values

	Daytime BP threshold (regardless of nighttime BP)			24‐h BP threshold (regardless of daytime or nighttime BP)		
Parameter	CH	NRMUCH	RMUCH	CH	NRMUCH	RMUCH
n.	523	178	37	463	231	44
Clinic SBP, mmHg	130 ± 7	134 ± 5*	134 ± 5*	129 ± 7	133 ± 6*	133 ± 7*
Clinic DBP, mmHg	80 ± 6	83 ± 5*	83 ± 6*	79 ± 6	83 ± 5*	83 ± 6*
Daytime SBP, mmHg	121 ± 8	137 ± 7*	138 ± 6*	121 ± 8	134 ± 8*	136 ± 8*
Daytime DBP, mmHg	75 ± 6	84 ± 7*	83 ± 8*	74 ± 6	83 ± 7*	82 ± 8*
Nighttime SBP, mmHg	110 ± 11	122 ± 12*	126 ± 11*	109 ± 9	122 ± 12*	126 ± 11*
Nighttime DBP, mmHg	65 ± 7	72 ± 8*	72 ± 9*	63 ± 6	73 ± 7*	73 ± 9*
24‐h SBP, mmHg	118 ± 8	133 ± 8*	134 ± 7*	117 ± 7	131 ± 8*	132 ± 8*
24‐h DBP, mmHg	72 ± 6	80 ± 7*	79 ± 8*	71 ± 6	80 ± 6*	79 ± 8*

BP, blood pressure; CH, controlled hypertension (below threshold value for each classification); DBP, diastolic blood pressure; NRMUCH, nonresistant masked uncontrolled hypertension (above threshold value for each classification); RMUCH, resistant masked uncontrolled hypertension (above threshold value for each classification); SBP, systolic blood pressure.

* *P* < .05 versus CH.

Antihypertensive therapy is shown in Table 3. Use of diuretics and calcium antagonists was higher in patients with RMUCH than in the other groups. Use of beta‐blockers, ace inhibitors, angiotensin receptor blockers, and alpha‐blockers was or tended to be higher in patients with RMUCH. Single, double, and triple therapy were different among the groups by definition.

**TABLE 3:** Antihypertensive therapy

	Daytime BP threshold (regardless of nighttime BP)			24‐hour BP threshold (regardless of daytime or nighttime BP)		
Parameter	CH	NRMUCH	RMUCH	CH	NRMUCH	RMUCH
n.	523	178	37	463	231	44
Diuretic, *n* (%)	207 (40)	51 (29)*	33 (89)*^†^	185 (40)	67 (29)*	39 (89)*^†^
Beta‐blocker, *n* (%)	177 (34)	51 (29)	16 (43)	163 (35)	61 (26)	20 (45)^†^
Calcium antagonist, *n* (%)	145 (28)	55 (31)	23 (62)*^†^	119 (26)	76 (33)	28 (64)*^†^
ACE‐I, *n* (%)	226 (43)	61 (34)	23 (62)^†^	202 (44)	81 (35)	27 (61)^†^
ARB, *n* (%)	105 (20)	24 (13)	12 (32)^†^	98 (21)	31 (13)*	12 (27)
Alpha‐blocker, *n* (%)	51 (10)	19 (11)	7 (19)	40 (9)	28 (12)	9 (20)*
Single therapy, *n* (%)	246 (47)	99 (56)	0 (0)*^†^	220 (48)	125 (54)	0 (0)*^†^
Double therapy, *n* (%)	194 (37)	79 (44)	0 (0)*^†^	167 (36)	106 (46)*	0 (0)*^†^
Triple therapy, *n* (%)	83 (16)	0 (0)*	37 (100)*^†^	76 (16)	0 (0)*	44 (100)*^†^

ACE‐I, angiotensin‐converting enzyme inhibitor; ARB, angiotensin receptor blocker; BP, blood pressure; CH, controlled hypertension (below threshold value for each classification); NRMUCH, nonresistant masked uncontrolled hypertension (above threshold value for each classification); RMUCH, resistant masked uncontrolled hypertension (above threshold value for each classification).

* *P* < .05 versus CH and ^†^ *P* < .05 versus NRMUCH for each classification.

Use of aspirin and statin was not different among patients with CH, NRMUCH, and RMUCH according to daytime threshold (15% vs. 11% vs. 22% and 9% vs. 6% vs. 5%, respectively). Use of aspirin was higher in patients with RMUCH than in those with NRMUCH according to 24‐h threshold (25% vs. 11%, respectively, *P* < .05). Use of statin was not different among patients with CH, NRMUCH, and RMUCH according to 24‐h threshold (9% vs. 6% vs. 4%, respectively).

**FIGURE 1:**
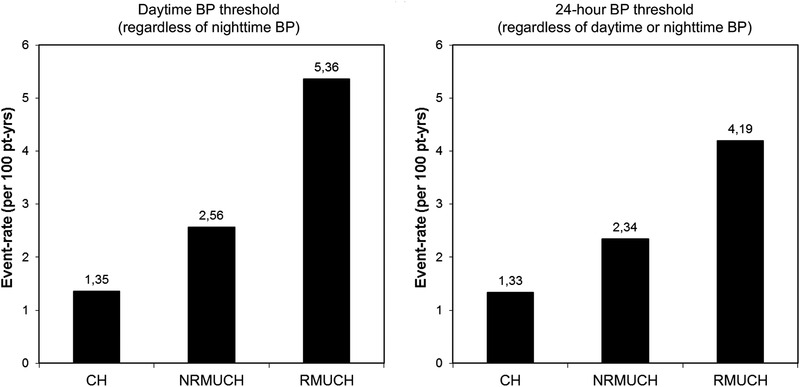
Event rates in patients with controlled hypertension (CH), nonresistant masked uncontrolled hypertension (NRMUCH), and resistant masked uncontrolled hypertension (RMUCH) defined according to daytime or 24‐h BP thresholds. There were 79, 52, and 17 events in patients with CH, NRMUCH, and RMUCH defined according to daytime BP threshold, respectively, and 67, 64, and 17 events in patients with CH, NRMUCH, and RMUCH defined according to 24‐h BP threshold, respectively. BP, blood pressure

**FIGURE 2:**
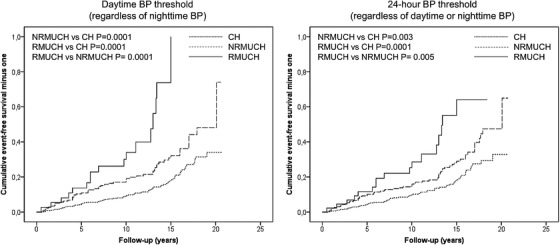
Event‐free survival curves of study groups. CH, controlled hypertension; NRMUCH, nonresistant masked uncontrolled hypertension; RMUCH, resistant masked uncontrolled hypertension

During the follow‐up (median 10 years, interquartile range 6–14 years), 148 events occurred in patients with CH, NRMUCH, and RMUCH. Event rate was progressively higher from CH to NRMUCH to RMUCH for each definition (Figure 1). Survival curves are reported in Figure 2.

Results of univariate and multivariate Cox regression analyses are reported in Table 4. Cardiovascular risk was higher in patients with NRMUCH than in those with CH and even higher in patients with RMUCH than in those with CH. This trend was observed in both unadjusted and adjusted analyses and according to both daytime and 24‐h BP thresholds, although the risk associated with subtypes defined according to daytime BP threshold tended to be higher. If components of the composite endpoint, that is, heart failure requiring hospitalization, coronary events and stroke were analyzed separately, the same trend was observed, though statistical significance was not always achieved because of the lower number of events for each category (Supplemental Table 1). When RMUCH was compared with NRMUCH, the risk tended to be higher in patients with RMUCH than in those with NRMUCH but statistical significance was not achieved (hazard ratio (HR) 1.65, 95% confidence interval (CI) .94–2.91, *P* = .08, and HR 1.40, 95% CI .81–2.42, *P* = .23, according to daytime and 24‐h BP thresholds, respectively).

**TABLE 4:** Risk of cardiovascular events in nonresistant and resistant masked uncontrolled hypertension when compared to controlled hypertension

	Daytime BP threshold (regardless of nighttime BP)		24‐h BP threshold (regardless of daytime or nighttime BP)	
	NRMUCH	RMUCH	NRMUCH	RMUCH
	HR (95% CI)	HR (95% CI)	HR (95% CI)	HR (95% CI)
Unadjusted	1.88 (1.32–2.66)	4.95 (2.92–8.38)	1.69 (1.20–2.38)	3.56 (2.09–6.07)
Adjusted^a^	1.81 (1.27–2.57)	2.99 (1.73–5.16)	1.58 (1.12–2.23)	2.21 (1.27–3.82)

Abbreviations: BP, blood pressure; CI, confidence interval; HR, hazard ratio; NRMUCH, nonresistant masked uncontrolled hypertension; RMUCH, resistant masked uncontrolled hypertension.

^a^ Adjusted for age, sex, body mass index, smoking habit, family history of cardiovascular disease, diabetes, previous events, estimated glomerular filtration rate < 60 mL/min, low‐density lipoprotein cholesterol, left ventricular hypertrophy, left atrial enlargement, asymptomatic left ventricular systolic dysfunction.

If clinic BP was forced into the models, if the few patients with RMUCH who did not take a diuretic were excluded, and if patients with CH were divided into those taking ≤2 or ≥3 drugs, results remained substantially similar (Supplemental Table 2).

When study groups were defined according to nighttime BP threshold (120/70 mmHg) regardless of daytime BP, RMUCH was not at significantly higher risk than CH and it was at slightly higher risk than NRMUCH (Supplemental Table 3). When CH, NRMUCH and RMUCH were defined by both daytime and nighttime BP thresholds, results tended to be similar to those obtained by using daytime BP threshold regardless of nighttime BP (Supplemental Table 3).

## 4 DISCUSSION

The present study shows that both NRMUCH and RMUCH are at increased cardiovascular risk than CH.

Previous studies showing higher cardiovascular risk in patients with MUCH than in those with CH have analyzed the MUCH group as a unique entity.^1^, ^2^, ^3^, ^4^, ^5^, ^6^, ^7^, ^8^, ^9^, ^10^, ^11^, ^12^, ^13^, ^14^, ^15^, ^16^ At present, to the best of our knowledge, only one study has evaluated the prognostic value of RMUCH.^19^ Barochiner and colleagues, by using home BP recording, studied 470 treated hypertensive patients who were receiving at least three antihypertensive drugs.^19^ Among individuals with controlled clinic BP, about one‐third had RMUCH.^19^ During a mean follow‐up of 6.7 years, patients with RMUCH were at higher risk of cardiovascular mortality (HR 4.9, 95% CI 1.2–19.9, *P* = .03) and of fatal and nonfatal cerebrovascular events (HR 5.1, 95% CI 1.5–16.9, *P* = .01) when compared to individuals with CH.^19^

At variance with the previous study,^19^ we used ambulatory BP monitoring to define study groups, included patients with NRMUCH and RMUCH and evaluated a composite cardiovascular endpoint. In our population, moreover, patients were about 10 years younger, a higher prevalence of men was observed in the RMUCH group, prevalence of diabetes and previous events was lower, and antihypertensive drug class distribution tended to be different in both patients with CH and in those with RMUCH. When single components of the composite endpoint were analyzed separately in our study, the same trend of all events was observed, though direct comparison with previous study^19^ is difficult because of different definition and evaluation of events. Some differences between our study and previous one^19^ could be partly explained by the above‐mentioned characteristics. In any case, results of both studies are essentially in line emphasizing the prognostic relevance of RMUCH. Our study adds a further information by showing the prognostic contribution of NRMUCH and RMUCH subtypes in the MUCH group.

After adjustment for various covariates, RMUCH tended to show higher risk than NRMUCH but statistical significance was not achieved. Daytime and nighttime systolic BP were 1–2  and 4 mmHg higher, respectively, in patients with RMUCH. When groups were defined by using nighttime BP threshold, RMUCH was not at significantly higher risk than CH and it was at slightly higher risk than NRMUCH. Thus, the aforesaid differences in BP may partly explain the tendency toward increased risk of RMUCH. It could be speculated that, in line with what happens in resistant hypertension, other factors beyond those considered in our analyses, such as aldosterone excess, a more severe degree of vascular dysfunction, vascular atherosclerosis, and undetermined features could contribute to explain the higher risk trend of RMUCH.^29^ However, whether or not RMUCH is at increased risk than NRMUCH and the potential underlying mechanisms require further investigation.

At present, there are not yet data showing the superiority of out‐of‐office BP control over clinic BP control in reducing risk and a multicenter study^30^ is ongoing to evaluate whether out‐of‐office BP control improves cardiovascular outcome in patients with MUCH. In any case, given the results of the present study, it could be suggested that therapeutic strategies should be properly addressed on MUCH subtypes for a better risk reduction.

The present study has some limitations. First, we studied only Caucasian patients and our results cannot be applied to other ethnic groups. Second, it was possible to evaluate adherence to therapy only in a part of the patients; in this context, however, there was no difference between those taking ≤2 or ≥3 drugs. Third, we did not specifically design a study to evaluate the risk associated with NRMUCH and RMUCH, but this study is part of a prospective assessment of the prognostic value of ambulatory BP parameters and other risk markers in our initially treated hypertensive patients.

In conclusion, our study shows that both NRMUCH and RMUCH are at increased cardiovascular risk than CH. In this context, our data suggest that therapeutic strategy should be specifically addressed in patients with NRMUCH and RMUCH for a better reduction of cardiovascular risk.

## AUTHOR CONTRIBUTIONS

Francesca Coccina wrote the paper, revised the manuscript critically for important intellectual content, and gave final approval of the version to be submitted and of the revised version. Anna M. Pierdomenico performed statistical analysis, revised the manuscript critically for important intellectual content, and gave final approval of the version to be submitted and of the revised version. Chiara Cuccurullo collected the data, revised the manuscript critically for important intellectual content, and gave final approval of the version to be submitted and of the revised version. Jacopo Pizzicannella collected the data, revised the manuscript critically for important intellectual content, and gave final approval of the version to be submitted and of the revised version. Maria T. Guagnano collected the data, revised the manuscript critically for important intellectual content, and gave final approval of the version to be submitted and of the revised version. Giulia Renda designed the study, revised the manuscript critically for important intellectual content, and gave final approval of the version to be submitted and of the revised version. Oriana Trubiani designed the study, revised the manuscript critically for important intellectual content, and gave final approval of the version to be submitted and of the revised version. Francesco Cipollone designed the study, revised the manuscript critically for important intellectual content, and gave final approval of the version to be submitted and of the revised version. Sante D. Pierdomenico collected the data, designed the study, contributed to the writing of the paper, revised the manuscript critically for important intellectual content, and gave final approval of the version to be submitted and of the revised version.

## Supporting information

Supporting information.
