# Supplementary material for: Prognostic value of non‐resistant and resistant masked uncontrolled hypertension detected by ambulatory blood pressure monitoring
Source: J Clin Hypertens (Greenwich). 2022 Mar 17;24(5):591–7. doi: 10.1111/jch.14460 (PMC9106087; doi:10.1111/jch.14460)
Supplement: Supplementary file 1 — Supporting information. [file JCH-24-591-s001.doc]

**Supplemental Table 1**.

Risk of heart failure, coronary events and stroke in non-resistant and resistant masked uncontrolled hypertension when compared to controlled hypertension

Daytime BP Threshold 24-hour BP Threshold

(regardless of nighttime BP) (regardless of daytime or nighttime BP)

NRMUCH RMUCH NRMUCH RMUCH

HR (95% CI) HR (95% CI) HR (95% CI) HR (95% CI)

| Heart Failure  Unadjusted | 2.32 (1.15-4.72) | 9.23 (3.61-23.6) | 2.10 (1.04-4.26) | 6.35 (2.44-16.6) |
| --- | --- | --- | --- | --- |
| Heart Failure  Adjusted^a^ | 2.40 (1.17-4.93) | 4.61 (1.71-12.5) | 2.13 (1.04-4.37) | 3.50 (1.29-9.55) |
| Coronary Events  Unadjusted | 1.80 (1.05-3.07) | 3.90 (1.63-9.31) | 1.71 (1.02-2.88) | 2.90 (1.20-7.00) |
| Coronary Events  Adjusted^a^ | 1.65 (0.96-2.82) | 2.35 (0.95-5.79) | 1.57 (0.93-2.65) | 1.81 (0.73-4.48) |
| Stroke  Unadjusted | 1.29 (0.67-2.49) | 2.95 (1.03-8.42) | 1.12 (0.60-2.08) | 2.08 (0.73-5.97) |
| Stroke  Adjusted^a^ | 1.38 (0.71-2.67) | 2.39 (0.82-7.00) | 1.08 (0.57-2.02) | 1.74 (0.60-5.10) |

BP, blood pressure; CI, confidence interval; HR, hazard ratio; NRMUCH, non-resistant masked uncontrolled hypertension; RMUCH, resistant masked uncontrolled hypertension. ^a^Adjusted for age, sex, body mass index, smoking habit, family history of cardiovascular disease, diabetes, previous events, estimated glomerular filtration rate < 60 mL/min, low density lipoprotein cholesterol, left ventricular hypertrophy, left atrial enlargement, asymptomatic left ventricular systolic dysfunction. In the study population there were 37 heart failures, 63 coronary events, 46 strokes, and 2 peripheral revascularizations.

**Supplemental Table 2**.

Risk of cardiovascular events in non-resistant and resistant masked uncontrolled hypertension when compared to controlled hypertension

Daytime BP Threshold 24-hour BP Threshold

(regardless of nighttime BP) (regardless of daytime or nighttime BP)

NRMUCH RMUCH NRMUCH RMUCH

HR (95% CI) HR (95% CI) HR (95% CI) HR (95% CI)

| Adjusted^a^  (+ clinic systolic BP) | 1.83 (1.28-2.61) | 3.00 (1.74-5.18) | 1.57 (1.11-2.22) | 2.20 (1.27-3.81) |
| --- | --- | --- | --- | --- |
| Adjusted^a^  (+ clinic diastolic BP) | 1.81 (1.27-2.58) | 3.00 (1.73-5.17) | 1.58 (1.11-2.23) | 2.21 (1.27-3.82) |
| Adjusted^a^  (excluding subjects not taking a diuretic) | 1.79 (1.25-2.55) | 2.90 (1.65-5.08) | 1.58 (1.11-2.23) | 2.39 (1.36-4.20) |
| Adjusted^a^  (versus subjects with CH taking < 2 drugs) | 1.75 (1.22-2.52) | 2.89 (1.66-5.03) | 1.56 (1.09-2.23) | 2.18 (1.25-3.81) |
| Adjusted^a^  (versus subjects with CH taking > 3 drugs) | 2.19 (1.11-4.32) | 3.61 (1.64-7.98) | 1.69 (0.86-3.32) | 2.37 (1.07-5.23) |

BP, blood pressure; CH, controlled hypertension; CI, confidence interval; HR, hazard ratio; NRMUCH, non-resistant masked uncontrolled hypertension; RMUCH, resistant masked uncontrolled hypertension. ^a^Adjusted for age, sex, body mass index, smoking habit, family history of cardiovascular disease, diabetes, previous events, estimated glomerular filtration rate < 60 mL/min, low density lipoprotein cholesterol, left ventricular hypertrophy, left atrial enlargement, asymptomatic left ventricular systolic dysfunction.

**Supplemental Table 3**.

Cardiovascular outcome in controlled hypertension and non-resistant and resistant masked uncontrolled hypertension defined by nighttime BP threshold (regardless of daytime blood pressure) and by daytime and nighttime blood pressure thresholds

Nighttime BP Threshold Daytime + Nighttime BP Threshold

(regardless of daytime BP)

CH NRMUCH RMUCH CH NRMUCH RMUCH

| n. | 381 | 297 | 60 | 339 | 140 | 33 |
| --- | --- | --- | --- | --- | --- | --- |
| Events, n | 55 | 75 | 18 | 43 | 42 | 15 |
| Event rate | 1.30 | 2.20 | 3.27 | 1.15 | 2.66 | 5.43 |
| Unadjusted  HR  (95% CI) | 1 | 1.68  (1.19-2.39) | 2.83  (1.66-4.82) | 1 | 2.31  (1.51-3.54) | 5.89  (1.27-10.6) |
| Adjusted^a^  HR  (95% CI) | 1 | 1.48  (1.04-2.11) | 1.67  (0.97-2.88) | 1 | 2.09  (1.36-3.22) | 2.92  (1.58-5.37) |

BP, blood pressure; CH, controlled hypertension; CI, confidence interval; HR, hazard ratio; NRMUCH, non-resistant masked uncontrolled hypertension; RMUCH, resistant masked uncontrolled hypertension. Daytime and nighttime BP thresholds are 135/85 and 120/70 mmHg, respectively. ^a^Adjusted for age, sex, body mass index, smoking habit, family history of cardiovascular disease, diabetes, previous events, estimated glomerular filtration rate < 60 mL/min, low density lipoprotein cholesterol, left ventricular hypertrophy, left atrial enlargement, asymptomatic left ventricular systolic dysfunction.
